# Expressing Double-Stranded RNAs of Insect Hormone-Related Genes Enhances Baculovirus Insecticidal Activity

**DOI:** 10.3390/ijms20020419

**Published:** 2019-01-18

**Authors:** Zheming Liu, Xiaofang Wang, Yan Dai, Xiaoli Wei, Mi Ni, Lei Zhang, Zhen Zhu

**Affiliations:** 1 State Key Laboratory of Plant Genomics and National Center for Plant Gene Research (Beijing), Institute of Genetics and Developmental Biology, The Innovative Academy of Seed Design, Chinese Academy of Sciences, Beijing 100101, China; liuzheming2011@genetics.ac.cn (Z.L.); scarlett1222@163.com (X.W.); ydai@genetics.ac.cn (Y.D.); xlwei@genetics.ac.cn (X.W.); fannimi2001@gmail.com (M.N.); 2 University of Chinese Academy of Sciences, Beijing 100049, China

**Keywords:** recombinant baculovirus, RNA interference, juvenile hormone, *Helicoverpa armigera*

## Abstract

Baculoviruses have already been used for insect pest control, but the slow killing speed limits their further promotion and application. Here we provide a strategy for improving baculovirus insecticidal activity using Helicoverpa armigera nucleopolyhedrovirus (HearNPV) to express double-stranded RNAs (dsRNAs) targeting cotton bollworm (*Helicoverpa armigera*) juvenile hormone (JH)-related genes. Droplet-feeding bioassays show that the 50% lethal concentration (LC_50_) values of recombinant baculoviruses expressing the dsRNA of JH acid methyl transferase gene (*HaJHAMT*) and the JH acid binding protein gene (*HaJHBP*) were 1.24 × 10^4^ polyhedral inclusion bodies (PIB)/mL and 2.26 × 10^4^ PIB/mL, respectively. Both were much lower than the control value (8.12 × 10^4^ PIB/mL). Meanwhile, the LT_50_ of recombinant baculovirus expressing dsRNA of *HaJHBP* was only 54.2% of the control value, which means that larval death was accelerated. Furthermore, the mRNA level of target genes was reduced in recombinant baculovirus-treated cotton bollworm larvae. Transcription of several key genes involved in hormone signaling pathways—for example, ecdysone receptor gene (*HaEcR*)—was also altered. This study establishes a new strategy for pest management by interfering with insect hormone-related gene expression via baculoviruses, and the engineered baculoviruses have great potential application in cotton production.

## 1. Introduction

Insect pests cause large crop losses worldwide through direct damage and the transmission of plant diseases [1]. To date, spraying chemical insecticides remains the most common method of pest control [2], but problems such as environmental pollution, effects on human health, and the development of resistance in target pests have followed [3]. Baculoviruses, which are arthropod-specific viruses, have been successfully applied for agriculture and forestry pest management. They are safe for people and wildlife because of their extremely narrow host range [4,5]. Baculoviruses can cause epidemics in insect populations and be effective in the environment for a long time [6]. In Latin America, baculoviruses as commercial pesticides have been successfully applied in integrated pest management programs for decades in soybean and vegetable fields, as well as apple and pear orchards [7]. However, the slow killing speed (ranging from five days to more than two weeks) largely hampers their application as an insecticide [8,9]. Fortunately, the genome sequences of some notable baculoviruses were determined, and many strategies have been developed to improve their killing action and stability through recombinant DNA technology [2,8]. For example, recombinant baculovirus expressing an insect-specific toxin from the scorpion *Androctonus australis* and straw itch mite *Pyemotes tritici* showed improved insecticidal activity [10,11].

Insect hormones play an important role in the regulation of insect growth, development, and reproduction. Insect hormone analogues, such as tebufenozide, have resulted in larval death due to the interruption of hormone-mediated cell or organ development [12]. There is considerable interest in the possibility of using insect hormones or hormone regulatory proteins in conjunction with microbial delivery systems as a means of controlling insect pests. Several hormone-related genes were used in genetically modified baculoviruses to enhance their insecticidal activity, including the genes of a diuretic hormone, eclosion hormone, prothoracicotropic hormone, and juvenile hormone (JH) esterase [13,14,15,16]. However, just a small part of these recombinant baculoviruses showed significant improvement over parent wild-type baculoviruses in pesticidal activity. Alternatively, the deletion of a nonessential baculovirus gene coding ecdysteroid UDP-glucosyltransferase (EGT) provided a beneficial effect on killing speed [17]. EGT catalyzes the conjugation of sugar molecules to ecdysteroids, thus preventing the ecdysteroid from crossing cellular membranes [18].

RNA interference, which triggers specific gene silencing through the delivery of homologous double-stranded RNA (dsRNA) fragments [19], is widely used in biological research and shows great application potential. In agriculture, there are several successful cases of insect pest control using RNA interference (RNAi) technology [20,21,22,23,24]. The first RNAi-based pest control agent, a transgenic corn that produces dsRNA and two *Bacillus thuringiensis* (Bt) toxins against the western corn rootworm, was approved for release in the field [25]. Baculoviruses have many advantages for RNAi delivery due to their inertness, versatility, and possibility of high throughput preparation [5]. Nevertheless, research on the application of baculovirus-mediated RNAi is mainly focused on gene therapy, and there have been few reports on pest control [26].

Cotton bollworm, *Helicoverpa armigera*, is one of the world’s most destructive pests in cotton and many other crops [27]. Helicoverpa armigera nucleopolyhedrovirus (HearNPV) has already been successfully applied to control cotton bollworms in Australia, the United States, China, and India [28]. However, since cotton bollworms do not die immediately after infection with HearNPV, it is difficult to effectively control pests and avoid losses in the outbreak period. In this work, we attempted to construct novel recombinant HearNPVs that can increase the insecticidal activity of HearNPV, and especially reduce the killing time. Our previous report showed that transgenic cotton expressing dsRNA targeting gene encoding proteins that synthesize or transport juvenile hormones was effective against cotton bollworm, especially for Bt-resistant cotton bollworm [24]. Insect resistance to the cotton-pyramiding Bt toxin and RNAi was substantially delayed compared to using Bt cotton alone. Here, to control cotton bollworm, we constructed two recombinant HearNPVs expressing dsRNA targeting JH acid methyltransferase gene (*HaJHAMT*) and JH-binding protein gene (*HaJHBP*) from *H. armigera*, and tested their insecticidal action. Our results demonstrate that the recombinant HearNPVs obtained in this study had significantly improved insecticidal activity.

## 2. Results

### 2.1. Construction of Recombinant Bacmid Containing dsRNA Expression Cassette

**Figure 1:**
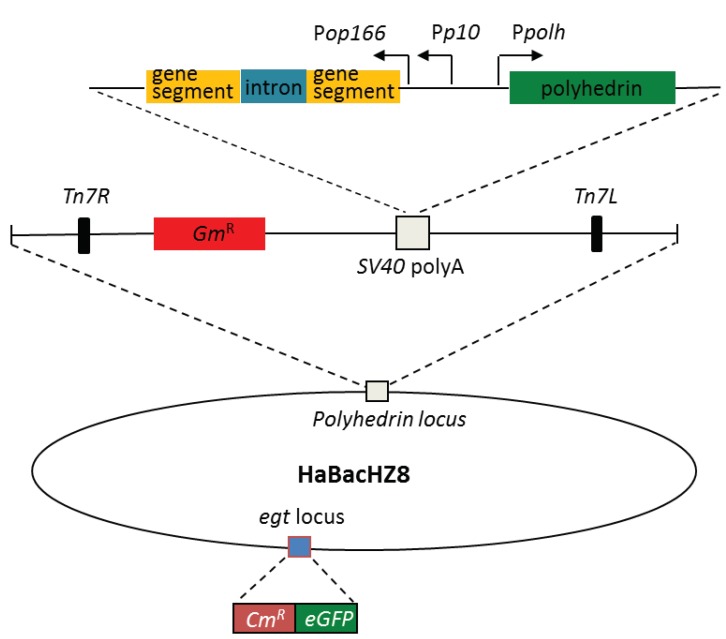
Schematic version of a recombinant baculovirus plasmid (bacmid). The hairpin RNA structure was composed of the same two juvenile hormone (JH)-related gene fragments in the antisense orientation separated by the intron from the potato GA20-oxidase gene. Double-stranded RNA (dsRNA) expression was controlled by the *op166* and *p10* promoters. The *polyhedrin* gene under its native promoter was reinserted in recombinant bacmids. In addition, the *egt* locus was replaced by *eGFP* and *Cm^R^* genes.

According to our previous report, transgenic cotton expressing dsRNA targeting *H. armigera* JH acid methyltransferase gene (*HaJHAMT*, GenBank accession number KX289532.1) or JH-binding protein gene (*HaJHBP*, GenBank accession number KX289533.1) showed resistance to cotton bollworm [24]. In this work, a 408 bp fragment of *HaJHAMT* and a 483 bp fragment of *HaJHBP* were used to compose hairpin RNAi constructs (see Supplementary Figures S1 and S2 for sequences). In order to obtain the recombinant baculovirus expressing dsRNA by the Bac-to-Bac system, the donor vectors, in which dsRNA expression was under the control of the *p10* promoter and *op166* promoter (Figure 1), were constructed and transformed into *E. coli* DH10B harboring HaBacHZ8Δegt and a helper plasmid. After site-specific transposition, we selected recombinant baculovirus plasmid (bacmids) based on white-blue screening, and further confirmed them by PCR. Validated bacmids were named HaBacΔegtdsJHAMT and HaBacΔegtdsJHBP.

### 2.2. Detection of Recombinant Baculoviruses in Transfected Cells

**Figure 2:**
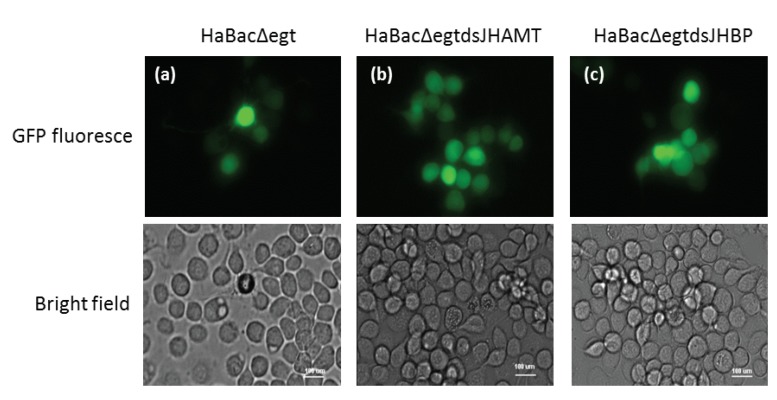
Microscopy observation of virus-transfected HzAM1 cells. HzAM1 cells were transfected with (**a**) HaBacΔegt; (**b**) HaBacΔegtdsJHAMT; or (**c**) HaBacΔegtdsJHBP. Pictures were taken three days post-transfection under ultraviolet light (**top** panels) or visible light (**bottom** panels). Bars = 100 µm.

To investigate the effect of the dsRNA insertion on virus infection and occlusion body (OB) formation, we transfected insect cells (HzAM1 cells), with HaBacΔegtdsJHAMT, HaBacΔegtdsJHBP, and HaBacΔegt (as a control). Since the *egt* gene locus of HaBacΔegt was replaced by the *eGFP* gene, green fluorescence should be observed in the infected cells. As expected, strong green fluorescence was observed under ultraviolet light in the HzAM1 cells at three days post-transfection (Figure 2). At the same time, a typical cytopathic effect, OB formation within the cells, was also observed on bright field.

**Figure 3:**
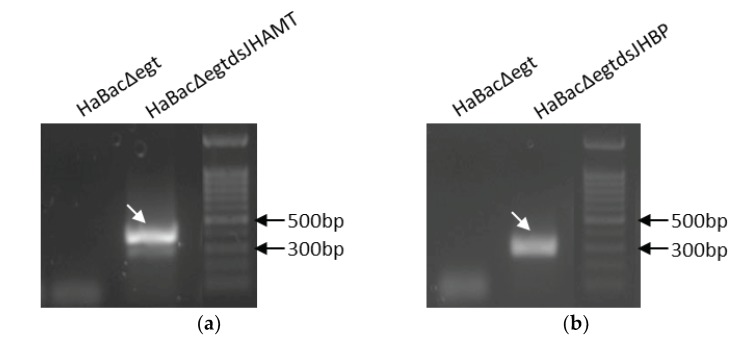
Transcription verification of dsRNA fragments in infected cells by reverse transcription (RT-PCR). (**a**) Detection of dsJHAMT total transcription in transfected insect cells; (**b**) Detection of dsJHBP transcription in transfected insect cells. HaBacΔegt-transfected insect cells were used as control. Total RNA of infected cells was extracted at 6 days after transfection. RT-PCR was performed and PCR products underwent electrophoresis in 1.5% agarose gel. Arrows point to target bands.

Furthermore, we extracted the total RNA of these infected cells and detected the transcription of dsRNA through reverse transcription PCR (RT-PCR). A 419-bp band of dsJHAMT-specific product was detectable in cells infected with HaBacΔegtdsJHAMT (Figure 3). Similarly, a 288 bp band of dsJHBP-specific product was also detected. No signal was seen in the control (cells infected with HaBacΔegt). These results confirmed again that the recombinants were constructed correctly and dsRNA was able to transcript as expected after infecting.

### 2.3. Efficacy of Recombinant Helicoverpa Armigera Nucleopolyhedroviruses in Killing H. armigera Larvae

To analyze the biological activity of recombinant HearNPV, we cultivated budded viruses (BVs) in HzAM1 cells and purified sufficient viral occlusion bodies from *H. armigera* larvae injected with BVs. Third instar *H. armigera* larvae were infected orally with selected doses of OBs and monitored for mortality. The 50% lethal concentration (LC_50_) value of vHaBacΔegtdsJHAMT was 1.24 × 10^4^ OBs/mL, which was just 15.3% of the control virus vHaBacΔegt value of 8.12 × 10^4^ OBs/mL (Table 1). Similarly, the LC_50_ value of vHaBacΔegtdsJHBP (2.26 × 10^4^ OBs/mL) was only 27.8% of the control value (Table 1). Statistical analysis indicated that the difference in the tLC_50_ value between vHaBacΔegtdsJHAMT and vHaBacΔegt was significant (*z* = 4.15, *p* < 0.05), as was the difference between vHaBacΔegtdsJHBP and vHaBacΔegt (*z* = 7.27, *p* < 0.05).

**Figure 4:**
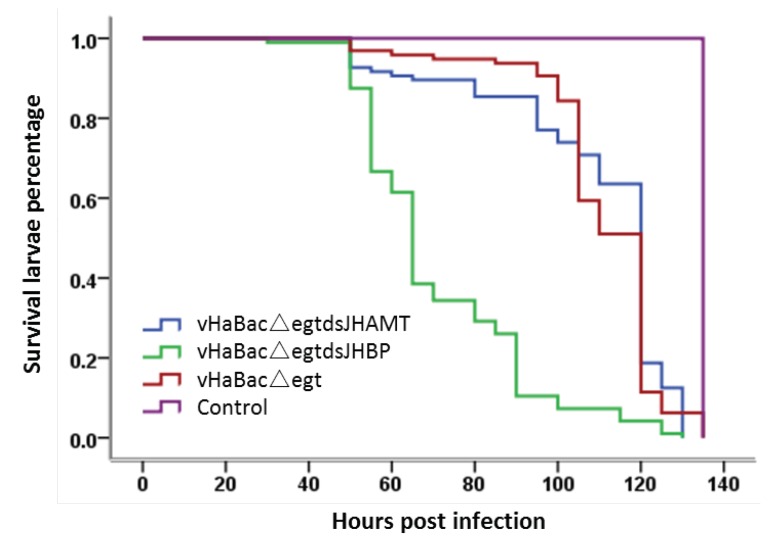
Survival analysis of third instar *H. armigera* larvae feeding with OBs of baculoviruses. The concentration of OBs was 100 times the LC_50_ value. The observation continued until all larvae died or pupated. Larvae feeding without baculovirus were used as controls; a total of 96 larvae were tested for each virus. Survival analysis were performed using the Kaplan–Meier estimator by SPSS 22.0.

Then we performed a survival analysis on the third instar *H. armigera* larvae that were fed HearNPVs. The virus concentration used in this experiment was 100 times the LC_50_, and the number of dead insects was recorded until all larvae died or pupated. The result showed that the survival time of larvae infected with vHaBacΔegtdsJHBP was greatly reduced (Figure 4), and the LT_50_ value of vHaBacΔegtdsJHBP (65 h) had a significant (χ^2^ = 111.7, *p* < 0.01) decrease of 45.8% compared to vHaBacΔegt (Table 2), while the survival time of larvae infected with vHaBacΔegtdsJHAMT was similar to that of vHaBacΔegt, as well as the LT_50_ values of vHaBacΔegtdsJHAMT (120 h) and vHaBacΔegt (120 h).

### 2.4. Downregulation of RNA Interference Target Gene Expression in Infected H. armigera Larvae

**Figure 5:**
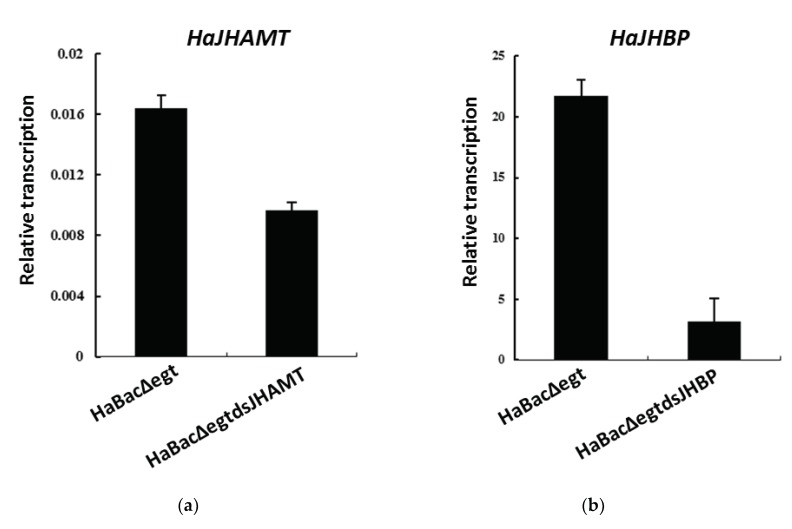
qRT-PCR analysis of RNA interference (RNAi) target genes in *Helicoverpa armigera* infected with vHaBacΔegtdsJHAMT, vHaBacΔegtdsJHBP, and vHaBacΔegt. (**a**) Detection of *HaJHAMT*; (**b**) Detection of *HaJHBP*. The cDNA from third instar larval at 48 h post infection were analyzed. *ACTIN* was selected as the reference gene. Data represent the ratio of gene expression to *ACTIN*. Bars show means and standard errors (three independent replicates).

We used quantitative real-time PCR (qRT-PCR) to detect the transcription level of RNAi target genes in *H. armigera* larvae that were infected by feeding with OBs for two days. The results showed that the mRNA level of *HaJHAMT* was suppressed in larvae infected with vHaBacΔegtdsJHAMT, in contrast to the control virus vHaBacΔegt (Figure 5a). Similarly, we found decreased expression of *HaJHBP* in larvae infected with vHaBacΔegtdsJHBP (Figure 5b).

### 2.5. Effect on Hormone-Related Downstream Genes in Infected H. armigera Larvae

**Figure 6:**
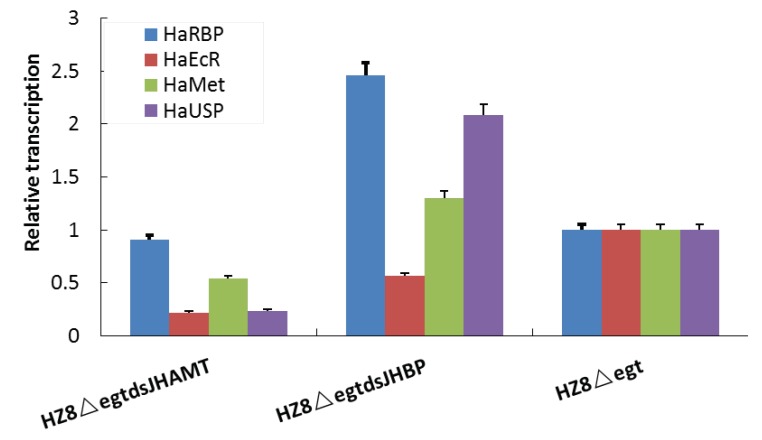
The qRT-PCR detection of genes involved in hormone pathways in third instar *Helicoverpa armigera* larvae. After 48 h of feeding with vHaBacΔegtdsJHAMT, vHaBacΔegtdsJHBP, and vHaBacΔegt, gene expression was analyzed by qRT-PCR. *ACTIN* was used as an internal standard to normalize the level of gene expression. Data of the histogram represents the ratio of gene expression to that in the control (vHaBacΔegt infected larvae). Bars show means and standard errors (three independent replicates).

To investigate the effect of recombinant HearNPVs on other hormone-related gene larvae, we detected mRNA levels of several key genes in orally infected *H. armigera* larvae using qRT-PCR, including the JH receptor gene *HaMet*, ultraspiracle (*HaUSP*), a potential candidate for the JH receptor, JH regulated gene RNA-binding protein (*HaRBP*), and ecdysone receptor gene *HaEcR*. We found that transcription of *HaRBP* and *HaUSP* increased in vHaBacΔegtdsJHBP-infected larvae compared to the control (vHaBacΔegt-infected larvae), while *HaUSP* expression was reduced in vHaBacΔegtdsJHAMT-infected larvae (Figure 6). Additionally, *HaEcR* expression decreased in both recombinant HearNPV-infected larvae.

## 3. Discussion

Baculoviruses are the most widely used virus insecticide in the world. However, in contrast to plant- or bacteria-mediated RNAi, the application of baculovirus as a dsRNA delivery vehicle for pest control has attracted far less attention [26]. In fact, it was verified 10 years ago that recombinant baculovirus expressing dsGFP can trigger RNAi and silence green fluorescence protein (GFP) in insect culture cells [29]. In this study, we successfully improved the insecticidal activity of baculovirus by expressing dsJHAMT and dsJHBP in it (Figure 1). Previous research indicated that baculovirus-mediated RNAi was not suitable for functionally characterizing genes of the lepidopteran insects, since the infection effects might mask the potentially produced phenotypes associated with RNAi [30]. Obviously, this phenomenon has no effect when baculovirus-mediated RNAi is applied to control pests.

The combined actions of JH and ecdysteroids are crucial in regulating insect molting and metamorphosis. The presence of JH during larval molting prevents pupation [31]. JHAMT is an irreplaceable enzyme for the unique reaction steps in juvenile hormone synthesis [32,33]. On the other hand, nearly every JH molecule binds to JHBP in hemolymph when transported from corpora allata cells, the JH synthesizing place, to its sites of action [34,35]. JHBP also protects the chemically labile JH from nonspecific enzymatic degradation [36]. Suppressing the expression of the two genes, *HaJHAMT* and *HaJHBP*, disturbs JH concentration in cotton bollworm and further affects larval survival [24]. Therefore, we chose to inhibit the expression of these two genes via baculovirus-mediated RNAi to improve the insecticidal activity of baculovirus.

In this study, we constructed two recombinant bacmids containing a dsJHAMT and dsJHBP expression cassette. To ensure that RNAi occurred in cotton bollworm as soon as possible after infection and persisted throughout the infection, dsRNA expression was driven by two baculovirus promoters, the early *op166* promoter and the late *p10* promoter (Figure 1). After HzAM1 cells were transfected with these recombinant bacmids, green fluorescence and OBs were observed in infected cells (Figure 2). These results indicate that the insertion of dsRNA did not affect virus infection and OB formation in HzAM1 cells. Further examination of infected cells by RT-PCR (Figure 3) confirmed the transcription of dsRNA as expected.

Bioassay results confirmed that our strategy to modify baculovirus was successful. Both recombinant HearNPVs in this study exhibited a large reduction in LC_50_ value compared to the control (Table 1), which shows that dsRNA expressed by baculoviruses played an extra insecticidal role in comparison with the control virus. However, for the LT50 bioassay, the result of vHaBacΔegtdsJHBP was significantly reduced, while there was no significant difference between vHaBacΔegtdsJHAMT and the control (Table 2). We suspected that this might be due to the lower virus concentrations used for infection than that of the control. The specific virus concentrations (100 times the LC_50_) were 2.26 × 10^6^ OBs/mL for vHaBacΔegtdsJHBP, 1.24 × 10^6^ OBs/mL for vHaBacΔegtdsJHAMT, and 8.12 × 10^6^ OBs/mL for the control, respectively. Further testing indicated that the transcription of dsRNA target genes *HaJHAMT* and *HaJHBP* in *H. armigera* larvae was suppressed in cotton bollworm larvae fed with recombinant OBs (Figure 5). This might reduce JH concentration in *H. armigera* [24]. Considering there was cross-talk between JH and ecdysteroid signaling [31], and that the transcription of other genes (*HaECR* et al.) involved in insect hormone signaling pathway was indeed altered at 48 h post-infection with recombinant HearNPVs (Figure 6), ecdysteroid function might also be affected in larvae. Therefore, we proposed a hypothesis. Recombinant HearNPVs could effectively express dsJHAMT and dsJHBP (Figure 3), and reduce target genes mRNA levels in infected larvae (Figure 5). This may lead to a decrease in JH concentration [24] and interfere with the hormonal equilibrium in infected host, which in turn might affect the growth and development of the larvae and ultimately results in larvae death. Obviously, this additional insecticidal effect will increase the insecticidal activity of recombinant HearNPVs. Specific to this study, it was reflected that recombinant HearNPVs had lower LC_50_ values and a shorter LT_50_ value than the control vHaBacΔegt (Table 1 and Table 2). In agreement with our results, recombinant baculoviruses expressing juvenile hormone esterase (JHE) or a mutant JHE (more stable in hemolymph than wild-type JHE) showed a dramatic reduction in LC_50_ in comparison to larvae infected with wild-type AcMNPV [16,37]. JHE is a catalytically inactive insect enzyme, and its overexpression decreases the concentration of JH [38]. Besides, the expression of dsJHBP rather than dsJHAMT could speed up the insecticidal activity of baculovirus in our research (Figure 4 and Table 2), possibly because JHBP is located farther downstream of the JH signaling pathway and has a faster impact on the JH titer than JHAMT.

In the past, three major approaches were pursued to improve the speed of baculovirus killing action by genetic engineering technology: (1) introduction of a gene encoding an insect-specific toxin, hormone, or enzyme into the baculovirus genome; (2) deletion of baculovirus genes that affect infection; and (3) incorporation of a toxin, like Bt toxin, into the polyhedrin matrix [2,39,40]. In this paper, we provide a new strategy: disturbing insect hormones by baculovirus-mediated RNAi. This strategy can be combined with previous improvements. For example, the baculovirus used in this study has already had the ecdysteroid UDP-glucosyltransferase (EGT) gene deleted, which may result in a reduction in food consumption and time to kill the host compared with wild-type HearNPV [41,42]. Laboratory analysis in this work has demonstrated that the recombinant HearNPV expressing dsJHBP could further shorten the killing time of *H. armigera* larvae with fewer occlusion bodies comparison to vHaBacΔegt. These characteristics make it possible for vHaBacΔegtdsJHBP to be further developed into a new bioinsecticide. However, we need to consider the production of baculoviruses for industrial application. The number of OB produced by each larva may be reduced, since larvae will die earlier after infection with vHaBacΔegtdsJHBP. Perhaps it would be more advantageous for industrialization to replace vHaBacΔegt with wildtype baculoviruses, which harbor the *egt* that prevents molting and prolongs the larval stage of infected insects, and allows for production of more progeny virus. Besides, as the recombinant baculovirus have stronger insecticidal activity, its application amount will also decrease.

## 4. Materials and Methods

### 4.1. Insects, Cell Line, and Viruses

*Helicoverpa armigera* larvae supplied by the Experimental Animal Center of Wuhan Institute of Virology, Chinese Academy of Sciences, were reared on an artificial diet at 26 °C, 60% humidity, and a 16 h/8 h photoperiod. *Helioverpa zea* cell line HzAM1 cells [43], kindly provided by Dr. Hu Zhihong (Wuhan Institute of Virology, Wuhan, China), were maintained in Grace’s insect medium supplemented with 10% FBS (fetal calf serum) at 28 °C. The infectious bacmid of HearNPV, HaBacHz8-Δegt (the *egt* locus was replaced by *eGFP* gene) [42], and intermediate plasmids pFBD-HaPH and pFB-op166 used in this study were also presented by Dr. Hu Zhihong [44,45].

### 4.2. Construction of Donor Plasmids

The baculovirus early promoter *op166* was amplified by PCR from plasmid pFB-op166 using primers OPF and OPR (see Supplementary Table S1). The obtained 184 bp amplicon was cloned into the pMD-18T vector for sequence verification. After digesting with *Nco*I and *Sma*I, *op166* promoter was inserted into the vector pFBD-HaPH downstream of the *p10* promoter to obtain the donor plasmid precursor pFBD-HaPH-op166. Hairpin RNAi constructs dsJHAMT and dsJHBP, as previously described [24], were excised by *Sse8387*I (blunting) from plasmids stored in our lab, and then cloned into the *Pvu*II enzyme site of pFBD-HaPH-op166 downstream of the *op166* promoter to produce donor plasmids pFBD-HaPH-op166-dsJHAMT and pFBD-HaPH-op166-dsJHBP, respectively.

### 4.3. Construction of Recombinant Bacmids

These two donor plasmids were transformed into *E. coli* DH10B-competent cells harboring a HaBacHz8-Δegt bacmid and helper plasmid expressing transposase, respectively. Correspondingly, the resulting recombinant bacmids produced by transposition were named HaBacΔegt-dsJHAMT and HaBacΔegtdsJHBP. According to the Bac-to-Bac^TM^ system manual, the colonies were selected in the presence of kanamycin, gentamicin, tetracycline, and X-Gal. White recombinants were picked and then checked by PCR using M13 forward primer and gene-specific primers.

### 4.4. Generation of Budded Viruses

Bacmid DNAs of HaBacΔegt-dsJHAMT, HaBacΔegtdsJHBP, and HaBacΔegt were purified from 5 mL cultures. A total of 5 μg bacmid DNA was used to transfect 5 × 10^5^ HzAM1 cells with the aid of 10 μL of lipofectin, following the manufacturer’s instructions (Invitrogen, Carlsbad, CA, USA). When cultured at 27 °C for three days, transfected cells were viewed for fluorescence under an inverted fluorescence microscope (Nikon TE-300, Tokyo, Japan). After six days’ culture, the cells were centrifuged at 4000 rpm for 10 min. The supernatant was collected as the first-generation virus and used to infect HzAM1 cells at a rate of 0.1 multiplicity of infection (MOI). When the yield of budded viruses (BVs) reached the peak (96 h), the supernatant, as the second-generation virus, was collected, and used to obtain the third-generation virus. The resulting BVs were named vHaBacΔegt-dsJHAMT, vHaBacΔegtdsJHBP, and vHaBacΔegt and tested for 50% tissue culture infective dose (TCID_50_) by endpoint dilution assay. All viruses were kept in the dark at 4 °C for further experiments.

### 4.5. Detection of dsRNA Transcripts in Infected Cells

To make sure the dsRNA expression cassette inserted into the HearNPV genome was active, reverse transcription PCR (RT-PCR) was used to detect dsRNA expression. The total RNA was extracted from infected HzAM1 cells at 6 dpi with Trizol (Invitrogen), according to the user manual. Next, 1.0 μg of total RNA was used to synthesize cDNA with a GoScript^TM^ reverse transcription system (Promega, Madison, WI, USA) and oligo(dT) anchor primer. Primer pairs JTF and Pin and Pin and JPR (see Supplementary Table S1) were used to detect the transcription of dsJHAMT and dsJHBP, respectively.

### 4.6. Purification of Viral Occlusion Bodies

Third instar *Helicoverpa armigera* larvae were infected via microinjection with the third-generation BVs (a titer of 1 × 10^7^ TCID_50_ mL^−1^, 5 μL for each larva), vHaBacΔegtdsJHAMT, vHaBacΔegtdsJHBP, and vHaBacΔegt. After about five days, cadavers of the larva that died from infection were collected and mashed. After adding some distilled water, the mixture was centrifuged at 300× *g* for 10 min to collect the supernatant. Then the supernatant was centrifuged again at 3000× *g* for 30 min. The precipitate was resuspended in distilled water and thoroughly mixed, which was the purified OBs. The purified OBs were diluted and counted under a microscope using a hemocytometer. Samples were counted three times and averaged.

### 4.7. Transcription Analysis of Genes in Infected H. armigera Larvae

The total RNA was extracted from three larvae that were infected with OBs and reared for two days. First-strand cDNA was synthesized by a GoScript^TM^ reverse transcription system (Promega) and diluted 10 times as a template. To measure transcription of *HaJHAMT*, *HaJHBP*, and related genes in OB-feeding *H. armigera* larvae, we performed quantitative real-time PCR (qRT-PCR) using a LightCycler 480 system (Roche, Basel, Switzerland) with LightCycler 480 SYBR Green I Master (Roche) following a two-step protocol: 95 °C for 10 min, 45 cycles of denaturation at 95 °C for 10 s, annealing at 58 °C for 20 s, and extension at 72 °C for 20 s. We used *ACTIN* as the reference gene, and calculated transcription relative to *ACTIN* with the 2^−ΔΔ*C*t^ method [46]. The primers used for qRT-PCR are listed in Supplementary Table S1.

### 4.8. Bioassays

LC_50_ values of recombinant HearNPVs were determined by the droplet feeding method [47]. Late second instar *H. armigera* larvae were placed in 24-well sterile plates and starved overnight at 28 °C. The following morning, larvae that had molted third instar were used for bioassays. The polyhedrosis virus was set to 3 × 10^5^ OBs/mL, 1 × 10^5^ OBs/mL, 3 × 10^4^ OBs/mL, 1 × 10^4^ OBs/mL, and 3 × 10^3^ OBs/mL suspensions. The starving third instar larvae were fed with the different suspensions of OBs mixed with 4% sucrose and 1 mg/mL blue dye (erioglaucine disodium salt, Sigma-Aldrich, St. Louis, MO, USA). The larvae whose midgut changed to blue in 10 min were transferred individually into a 24-well plate containing fresh artificial diet. The mortality rate was observed twice a day until all larvae died or pupated. Each OB suspension concentration was tested using 24 larvae and the experiment was performed twice. The LC_50_ values were analyzed by Probit regression analysis and compared by *z*-test in SPSS (IBM SPSS Statistics 22.0, Armonk, NY, USA).

LT_50_ values of recombinant HearNPV were also tested by a droplet feeding method at a high OB concentration (100 × LC_50_). Second instar *H. armigera* larvae were infected as mentioned above. Time 0 was defined as the point where larvae were transferred to the fresh diet. Then we observed and recorded the number of deaths at 07:00, 13:00, 18:00, and 23:00 every day until all the larvae died or pupated. Each HearNPV was tested using 96 larvae. LT_50_ values were calculated using the Kaplan–Meier estimator and further compared by a log-rank test (Mantel–Cox) in SPSS 22.0 [48].

## 5. Conclusions

In conclusion, the results obtained show that recombinant HearNPV expressing dsJHAMT or dsJHBP successfully suppressed target gene expression in infected cotton bollworm, and these recombinant HearNPVs had higher insecticidal activity than the unmodified baculovirus. Especially for vHaBacΔegtdsJHBP, the insecticidal speed increased by 45.8%, while the toxicity more than doubled. These data show the possibility for applying engineered baculoviruses in cotton bollworm control, and this research provides a new strategy for pest management by interfering with insect hormone-related genes via baculovirus-mediated RNAi.

## 6. Patents

Two patents resulted from the work reported in this paper. The patent authorization numbers are ZL201210576020.4 and ZL201210576007.9.

## Figures and Tables

**Table 1:** The 50% lethal concentration (LC_50_) values for recombinant Helicoverpa armigera nucleopolyhedroviruses (HearNPVs) in third instar *Helicoverpa armigera*.

Virus	LC_50_ (×10^4^ OBs/mL)	SE	95% Confidence Limit	
			Upper Bound	Lower Bound
vHaBacΔegtdsJHAMT	1.24	0.49	2.42	0.49
vHaBacΔegtdsJHBP	2.26	0.81	4.21	1.05
vHaBacΔegt	8.12	1.16	10.7	6.16

LC_50_: 50% lethal concentration; OBs: occlusion bodies; HearNPV: Helicoverpa armigera nucleopolyhedrovirus; SE: standard error.

**Table 2:** LT_50_ values for recombinant HearNPVs in third instar *Helicoverpa armigera*.

Virus	LT_50_ (Hours Post Infection)	SE	95% Confidence Limit	
			Upper Bound	Lower Bound
vHaBacΔegtdsJHAMT	120	0.89	118.26	121.74
vHaBacΔegtdsJHBP	65	1.08	62.88	67.12
vHaBacΔegt	120	1.02	118.01	122.00

Median lethal time value was calculated using the Kaplan–Meier estimator and further compared using a log-rank test (Mantel–Cox) by SPSS 22.0. SE: standard error.

## Supplementary Materials

Supplementary materials can be found at http://www.mdpi.com/1422-0067/20/2/419/s1.

## Abbreviations

bacmid	baculovirus plasmid
Bt	*Bacillus thuringiensis*
*eGFP*	enhanced green fluorescence protein gene
*Gm^R^*	gentamicin resistance gene
*Cm^R^*	chloramphenicol resistance gene
TCID_50_	50% tissue culture infective dose
PIB	polyhedral inclusion body
UDP	uridine diphosphate
