# Supplementary material for: Expressing Double-Stranded RNAs of Insect Hormone-Related Genes Enhances Baculovirus Insecticidal Activity
Source: Int J Mol Sci. 2019 Jan 18;20(2):419. doi: 10.3390/ijms20020419 (PMC6359566; doi:10.3390/ijms20020419)
Supplement: Supplementary file 1 [file ijms-20-00419-s001.pdf]

1 GAAAATAAAT AACAAGAATC ATAAACTAAA CAAATCAATA TCACGAAATT TGAACATCTG
61 TAACTAAAAA TATTTTCTTT AAAATGAATA ACGCGGTCTT GTATGAGAAA AGCAATAGCT
121 TGCAGAAGAG AGATGCTATC ATGTGTCTAG AAGAATACGC TTCGAAAATT AAGTGGAAGA
181 AGAGTAATAA TAATATTCTT GACATAGGCT GTGGGGATGG AAGCGTGACT AACATGCTGA
241 AGAAATATAT CCCTACTGAG TATAAGTTGC TTGGCTGTGA TATTAGCGAG AAGATGGTGA
301 ACTTCGCGAA TGATCATCAT TGTAACGAAC AGACTTCTTT CACCGTGCTT GATATCGAGG
361 GAGATCTACC TGAAGGCATG AAGGGAAACT TCGACCACGT ATTCTCGTTC TACGCTCTGC
421 ACTGGGTTAA TAACCAAGAA CGAGCATACA ACAACATATA CAACCTTCTA AGCGAGGATG
481 GAGAATGTTT CACGATATTC GTGGCATGGG CTCCTGTGTT TGACGTGTAC CGGGTACTCG
541 CGCGCAACAA CAAGTGGAGT CAATGGGTGC ATGATGTCGA CAGATACATA TCACCGTACC
601 ACGACTCGTT GGAGCCTGAA AAAGATTTAA AGACAATGAT AGACAAAATT GGATTCGTTG
661 ACGTCGACGT GGAATGCAAA GAATTGGTAT TCGTGTATGA CAACATACAT ATTTTGCGAA
721 AAGCGCTAAC AGCAATCAAC CCTTTCAAAA TTCCCAAGGA AAAATATGAT GACTTCATGG
781 AAGATTACAT GGACATATTG AAAGAACTAC AGATCTTAGA CAAGTACAAC AATAATTATG
841 AAAAGAGCGT TGAATTCAAT TACCGTTTGC TTGTAGTGTA TGCTCGGAAG CCTGACTCGC
901 AGGATAAAAC GTTAGAAGCA CTAAATGGGC AGACGTAGGT ATTTTAAATA TACATATATC
961 TATGGTAAAA CACGGTGTAA AACAGTTATT AGTAGTTAAG GATGAATGTA TAGTGTATCT
1021 CTTCAGGTTT AGTTTT

Figure S1. mRNA sequence of *HaJHAMT* (GenBank accession number KX289532.1). Fragment used as dsJHAMT was underlined.

1 AAGACTACAA CATGGCAGTT TATAGGAGCT TGATATTGCT TGCATTTGCG AGTTGTGTAC
61 TTTCGGAAGG AGGAACACTT TTCAATCCAT GCAGTAAAAA TGACATAAAA TGCTTGAGTG
121 GAGCAACCGA ATCTTTTTTG GAAAAAACTA GCAATGGTTT TCCGGATTAC AAAATCAAGG
181 CTATTGACCC TTTGATCATT CCTGAGTTAA AAGTCGTGGT CGACGAAGGC TTGGGACTGG
241 TCTATGATTT CAAAAACATA AATATAACTG GACTGAAGAC GCAGCAGATA TCAGACTTCA
301 AAATGGATAC AGACAAGAAA TCTGTGGTTT TAAAAACAAA AGCTGTTTTG AATATCGTGG
361 GTGATGTCAA AATCGAATTC GCCAAGCAAA ATAAAGTTTT TAATGGAGCT TATACAGCTT
421 CAACAACTGC TATAGGAAGC TCACAATATG GCTACAGTTT TAAGAAAAAA GATGATAAAG
481 ATCACTTCGT AGTCGGCCCA GAGGTGAATA CATGTGAAAT CATTGGAGAA CCGAACGTGG
541 ATATTGGAGA TGATTTACAA AAGGCCTTGG ACAGTGACTC CGATGCACAG GCTCTGAAAC
601 CTGATTACGA GACTAACAAG GTGGCTTTAC GGAAGAAGAC GCTATGCCAT ATTGTAGAAG
661 CCGCCTACGT CACCGTCATA CACAACATCA GGGCAATCGC AGATATCTTC CCTAAGGAAG
721 CCTTTTTCAC TGACATTTAA AAGCCGCCTA CGTCACCGTC ATACACAACA TCAGGGCAAT
781 CGCAGATATC TTCCCTAAGG AAGCCTTTTT CACTGACATT TAATA

Figure S2. mRNA sequence of *HaJHBP* (GenBank accession number KX289533.1). Fragment used as dsJHBP was underlined.

Table S1. Primers used in this study.

Primer	Sequence	Amplification Purpose
OPF	5′-CCATGGGGTCTTGTAGGTCTTGTAGT-3′, *NcoI* underlined	Primers for amplification of *OP166* promoter
OPR	5′-CCCGGGCGGAAATTATCGCAAGATAAGG-3′, *SmaI* underlined	
Pin	5′-GAAATTTTTGAATCTTGCAGTCAGC-3′	Primers for the detection of dsJHBP
JPR	5′-TTCGTAGTCGGCCCAGAGGT-3′	
JTF	5′-CAACGAGTCGTGGTACGGTGAT-3′	Primers for the detection of dsJHAME
Pin	5′-GAAATTTTTGAATCTTGCAGTCAGC-3′	
P20-F	5′-ATTGCTTGCATTTGCGAGTTGTGT-3′	*HaJHBP* specific primers for qRT-PCR
P20-R	5′-TCCGGGAAACCATTGCTTGT-3′	
P35-F	5′-GGGTACTCGCGCGCAACAACAA-3′	*HaJHAMT* specific primers for qRT-PCR
P35-R	5′-ATCCTGCGAGTCAGGCTTCCG-3′	
P41F	5′-AGGAAGCACAGGAAGAGGA-3′	*HaRBP* specific primers for qRT-PCR
P41-R	5′-AAGCACCAGTCCACGGAAA-3′	
P42-F	5′-AATTGCCCGTCAGTACGA-3′	*HaECR* specific primers for qRT-PCR
P42-R	5′-TGAGCTTCTCATTGAGGA-3′	
P43-F	5′-ATCCAAGCCACTCACAGC-3′	*HaMET* specific primers for qRT-PCR
P43-R	5′-AGCGGCAAGTCTCAACAC-3′	
P44-F	5′-GGTCCTGACAGCAATGTT-3′	*HaUSP* specific primers for qRT-PCR
P44-R	5′-AGCTCCAGCTGACTGAAG-3′	
P21-F	5′-CCTGGTATTGCTGACCGTATGC-3′	*HaACTIN* specific primers for qRT-PCR
P21-R	5′-CTGTTGGAAGGTGGAGAGGGAA-3′	
